# Paradoxical Inflammatory Bowel Disease Induced by Golimumab in a Patient With Ankylosing Spondylitis: A Case Report and Systematic Review

**DOI:** 10.7759/cureus.77363

**Published:** 2025-01-13

**Authors:** Khalid A Alnaqbi, Amna Riaz, Mohammed Alaswad

**Affiliations:** 1 Department of Research, Emirates Medical Association, Dubai, ARE; 2 Department of Internal Medicine, College of Medicine and Health Sciences, United Arab Emirates (UAE) University, Al Ain, ARE; 3 Division of Rheumatology, Sheikh Tahnoon Medical City, Al Ain, ARE; 4 Division of Rheumatology, Tawam Hospital, Al Ain, ARE; 5 Department of Internal Medicine, Sheikh Tahnoon Medical City, Al Ain, ARE; 6 Department of Internal Medicine, Tawam Hospital, Al Ain, ARE; 7 Faculty of Medicine, University of Hama, Hama, SYR

**Keywords:** ankylosing spondylitis, axial spondyloarthritis, golimumab, ibd-associated arthritis, ibd-related arthritis, inflammatory bowel disease, paradoxical reaction, seronegative arthritis, systematic literature review, tumor necrosis factor-alpha inhibitor

## Abstract

Paradoxical reactions (PRs) to biologic medications, such as psoriasis, arthritis, and inflammatory bowel disease (IBD), have been increasingly recognized. The aim of reporting this case is to establish an association between golimumab and exacerbation or new (de novo) IBD in patients with axial spondyloarthritis (SpA).

Our case involves a young patient with juvenile-onset ankylosing spondylitis (AS) who developed de novo IBD following golimumab therapy for active spinal disease. The patient had no prior gastrointestinal (GI) symptoms, and AS symptoms significantly improved with golimumab. However, before the third dose, he experienced non-bloody diarrhea, mild abdominal cramping, and constitutional symptoms (fever, chills, and weight loss). Colonoscopy and biopsy confirmed unclassified IBD. The discontinuation of golimumab resulted in marked improvement in GI symptoms, but the recurrence of AS symptoms necessitated the initiation of infliximab, which resolved both AS and IBD symptoms.

A comprehensive systematic literature review was conducted (from 2008 to October 2024) on Medical Literature Analysis and Retrieval System Online (MEDLINE) Complete/PubMed and Scopus databases using both Medical Subject Heading (MeSH) terms and keywords related to golimumab, SpA, and paradoxical IBD. Data from included cases were extracted by two researchers, and the quality assessment of case reports was performed using a standardized tool. Four cases of paradoxical IBD development following golimumab treatment in patients with pre-existing IBD were identified.

This is the first reported case of de novo IBD development in a biologic-naïve patient with AS treated with golimumab. This case highlights the importance of prompt evaluation of gastrointestinal symptoms and early gastroenterology referral during biologic therapy.

## Introduction

Ankylosing spondylitis (AS) is a prototype of a group of idiopathic chronic autoimmune inflammatory diseases known as spondyloarthritis (SpA). Symptoms encompass inflammatory back pain, peripheral arthritis, enthesitis, and dactylitis. Extra-musculoskeletal manifestations (EMM) include uveitis, psoriasis, and inflammatory bowel disease (IBD). While the pathogenesis of spinal inflammation in AS remains unclear, tumor necrosis factor (TNF)-α plays a role in the pathogenesis, as evidenced by the efficacy of TNF inhibitors, namely, infliximab, etanercept, adalimumab, golimumab, and certolizumab. The 2022 Assessment of SpondyloArthritis International Society and the European Alliance of Associations for Rheumatology (ASAS-EULAR) recommendations for the pharmacological management of axial spondyloarthritis (axSpA) include nonsteroidal anti-inflammatory drugs (NSAIDs), TNF inhibitors, IL-17 inhibitors, and Janus kinase (JAK) inhibitors [1].

TNF-α involvement in the pathogenesis of IBD is reflected in local cytokine expression in the gut tissue, as well as the therapeutic efficacy of anti-TNF agents for both Crohn's disease (CD) and ulcerative colitis (UC) [2]. Golimumab is a fully humanized monoclonal anti-TNF antibody that is approved for use in axial SpA (AS and non-radiographic axial SpA), psoriatic arthritis (PsA), rheumatoid arthritis (RA), UC, polyarticular juvenile idiopathic arthritis (JIA), and PsA in pediatric patients [3]. Biologic medications are commonly used for the treatment of many autoimmune diseases. Paradoxical reactions (PRs) involve a new or worsening condition that typically should improve when treated with the same agent that caused it. Such reactions can include arthritis, psoriasis, and IBD, particularly with TNF inhibitors [4-6].

Our case describes a paradoxical new-onset IBD in an AS patient shortly after the initiation of golimumab therapy. By sharing this unique case, we aim to raise awareness among clinicians about this rare adverse effect and contribute to the growing body of knowledge on paradoxical reactions to biologic agents.

## Case presentation

An 18-year-old Caucasian man was diagnosed with juvenile-onset AS at the age of 15 years (in 2008) based on inflammatory back pain, which started several months earlier, and radiographic evidence of bilateral sacroiliitis. The human leukocyte antigen B27 (HLA-B27) test was negative. His maternal grandmother and maternal grandfather had AS and RA, respectively. He had no history of cigarette smoking or alcohol consumption. In 2008, he was treated with oral naproxen 500 mg twice a day with clinical improvement, but this was discontinued after one year given persistent cough and wheezing. In 2009, an MRI revealed erosive changes in the right femoral head.

In September 2010, he presented with severe right hip and buttock pain of one-week duration and was found to have elevated C-reactive protein (CRP) at 13.7 mg/L (normal range < 5) and elevated erythrocyte sedimentation rate (ESR) at 27 mm/hour (normal range < 20 mm/hour). The pediatric rheumatologist recommended sulfasalazine; however, the patient and his parents declined. No evidence of EMM was found.

In April 2011, his care was transferred to an adult rheumatologist. He complained of bilateral shoulder pain associated with a 15-minute morning stiffness. On physical examination, his vital signs were normal, and his body mass index was 17.6 kg/m^2^. Musculoskeletal examination revealed right hip tenderness on internal rotation. Otherwise, it was normal including spinal mobility measurement. No enthesitis, dactylitis, or psoriasis was observed. His CRP level was elevated at 16 mg/L. Table 1 shows normal routine hematology and biochemistry tests, with negative hepatitis C and B serology, antinuclear antibodies, and rheumatoid factor.

**Table 1:** Investigations of our patient

	April 2011	May 2011	August 2011	Normal value
Hemoglobin, g/dL	13.3	-	14.2	14.0-18.0
Hematocrit, %	40.0	-	41.0	42.0-54.0
Mean corpuscular volume, fL	93.0	-	93.1	80.0-95.0
Platelets, cells/mm^3^	350	-	322	150-400
White cell count, cells/mm^3^	7.0	-	6.5	4.0-11.0
Neutrophils, cells/mm^3^	2.53	-	2.52	2.0-7.5
Lymphocytes, cells/mm^3^	1.80	-	1.99	1.50-4.00
Monocytes, cells/mm^3^	0.70	-	1.62	0.20-0.80
Eosinophils, cells/mm^3^	0.31	-	0.28	0.04-0.40
Basophils, cells/mm^3^	0.04	-	0.05	≤0.10
Alanine aminotransferase, IU/L	30	-	23	7-40
Aspartate aminotransferase, IU/L	31	-	20	5-34
Alkaline phosphatase, IU/L	100	-	90	40-150
Total bilirubin, mg/dL	0.5	-	0.4	≤1.3
Random blood glucose, mmol/L	6.0	-	-	3.9-7.8
Albumin, g/dL	4.1	-	4	3.8-5.0
C-reactive protein, mg/L	16	-	38	≤11.0
Erythrocyte sedimentation rate, mm/hour	20	-	18	0-10
Creatinine, mg/dL	-	-	1.10	0.72-1.44
Antinuclear antibody screen	Negative	-	-	Negative
Rheumatoid factor, IU/mL	<10	-	-	≤11
Hepatitis B surface antigen, surface antibody, and core antibodies	Negative	-	-	Negative
Hepatitis C antibody screen	Negative	-	-	Negative
Tuberculin skin test	-	Negative	-	Negative
Stool cultures for parasites, *Salmonella*, *Shigella*, *Yersinia*, and *Campylobacter* species	-	-	Negative	Negative
Stool smear for white blood cells	-	-	Positive	Negative
Assay for *Clostridium difficile* toxin	-	-	Negative	Negative

X-rays revealed bilateral grade 3 sacroiliitis and mild bilateral hip joint space narrowing, fulfilling the modified New York classification criteria for AS. His Bath Ankylosing Spondylitis Disease Activity Index (BASDAI) score was 2.3/10, indicating inactive disease, and his Bath Ankylosing Spondylitis Functional Index (BASFI) score was 0.1/10, indicating minor functional limitation. He was prescribed celecoxib 100 mg twice a day, which was discontinued at the end of May 2011 owing to inefficacy. Chest X-rays were normal, and the tuberculin skin test was negative.

On June 15, 2011, due to a flare of inflammatory back pain associated with a high CRP level, he was started on his first TNF inhibitor, golimumab, 50 mg/month subcutaneously. At that time, he did not report any symptoms suggestive of IBD, such as frequent diarrhea with urgency, mucus in the stool, abdominal cramping, or melena. One week after the first golimumab injection, his musculoskeletal symptoms improved significantly, allowing him to discontinue celecoxib. One week prior to the third injection, he developed intermittent low-grade fever (39°C) accompanied by chills, mild abdominal cramping, weight loss of 2.5 kg, and non-bloody, non-nocturnal watery diarrhea occurring four times a day. There was no associated steatorrhea, anorexia, jaundice, nausea, or vomiting. There has been no sick contact or travel during the past year. He had no recent history of antibiotic use or other conditions known to alter the gut microbiome. He did not consume raw seafood, raw eggs, undercooked meat or poultry, or unpasteurized dairy products. No cutaneous, neurological, or respiratory symptoms were observed. Symptoms of his AS were quiescent.

On August 15, 2011, he presented to the clinic with persistent gastrointestinal (GI) symptoms after taking the third injection. On physical examination, he appeared well, with a weight of 54 kg (a decrease of 2.5 kg compared to four months ago). Skin, nails, and abdominal examination were normal, with no lymphadenopathy, enthesitis, tenosynovitis, or dactylitis. Table 1 shows normal CBC, liver enzymes, albumin, and serum creatinine levels. CRP level increased to 38 mg/L, while ESR remained stable at 18 mm/hour. The infectious workup was negative for ova, parasites, and bacteria. Tests for enterovirus and fecal calprotectin were not performed. The stool smear was positive for white blood cells. Golimumab was withheld, and the patient was referred to a gastroenterologist.

**Figure 1:**
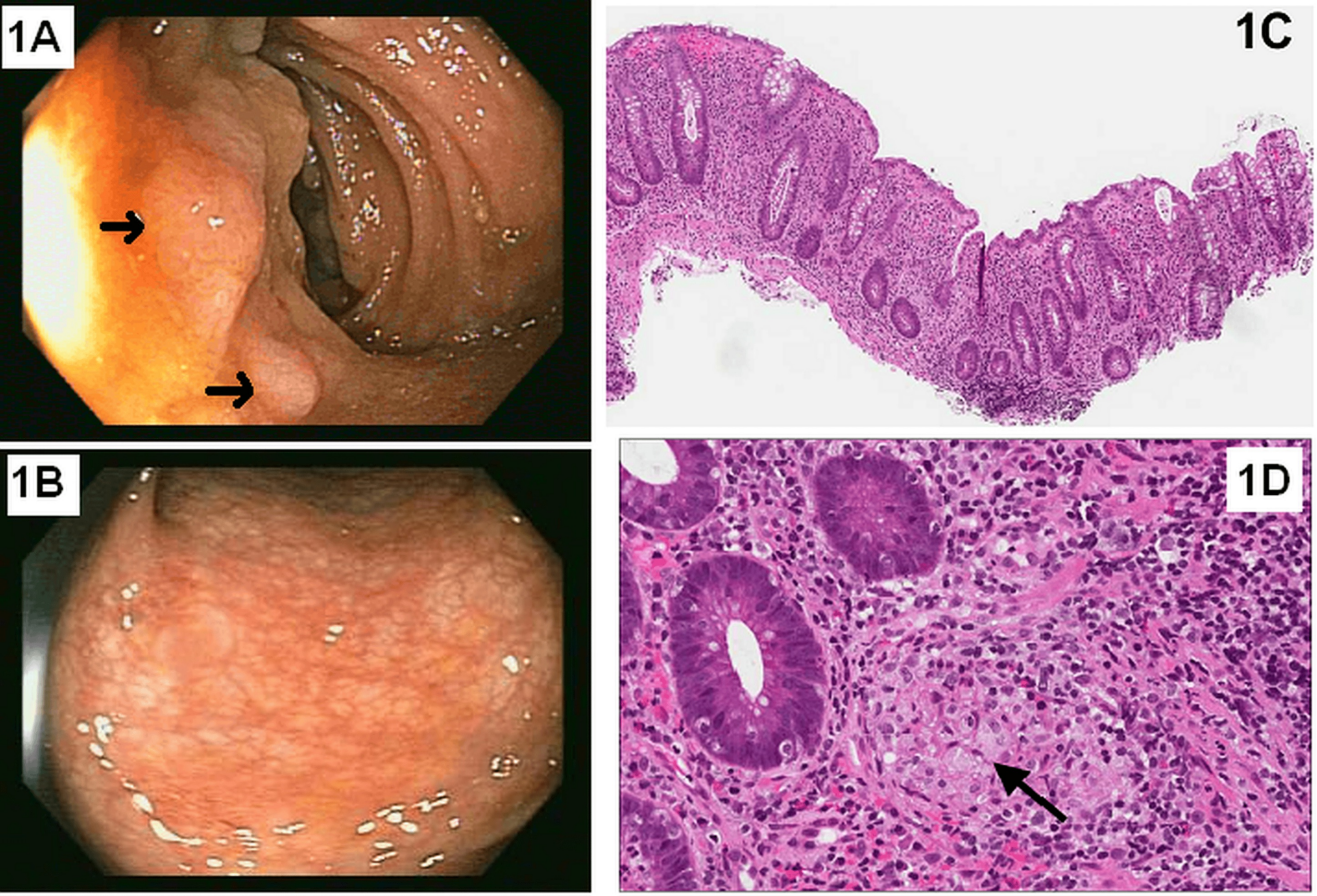
Colonoscopy and histology of the biopsy (A) Colonoscopic view showing mild fine nodularity (arrows) of the distal terminal ileum. (B) Colonoscopic view showing fine granularity, corkscrew vasculature, and mild erythema. (C) Diffuse chronic active colitis. (D) Rare histiocytic non-caseating granulomata in the ascending colon biopsy (arrow)

In December 2011, due to ongoing GI symptoms and intermittent fever, he underwent ileocolonoscopy, which revealed fine nodularity of the distal terminal ileum and mild pancolitis (Figure 1A and Figure 1B). Endoscopic findings revealed features of IBD. Multiple biopsies confirmed mild active ileitis and diffuse chronic active pancolitis (the cecum, ascending colon, transverse colon, descending colon, sigmoid colon, and rectum) with non-caseating granulomas (Figure 1C and Figure 1D).

The final pathological diagnosis was unclassified chronic active colitis (IBD unclassified, IBDU). Upon further questioning, the patient found out that one of his family members had CD.

The patient's bowel symptoms improved significantly three weeks after the cessation of golimumab treatment, and he regained his weight. His CRP level was normalized. His AS symptoms had remained quiescent until February 2012 when his disease flared (BASDAI score increased from 0.6/10 to 5/10), requiring him to restart celecoxib. At that time, flexible sigmoidoscopy was performed, which showed quiescent colonic disease. Infliximab (Remicade) 5 mg/kg intravenously was prescribed to treat his AS flare. After receiving infliximab infusion at zero and two weeks, his BASDAI score decreased to 0.7/10, with a complete resolution of his bowel symptoms. Symptoms of AS and IBDU remained quiescent at two years of follow-up until he was lost to follow-up.

## Discussion

We performed a systematic literature review to identify any related cases or reports similar to our case report.

Systematic review

Search Strategy and Study Selection

Two independent researchers (KAA and MA) conducted a comprehensive search of the Medical Literature Analysis and Retrieval System Online (MEDLINE) Complete/PubMed and Scopus databases, covering the literature from 2008 to October 9, 2024. Both Medical Subject Heading (MeSH) and non-MeSH keywords were used to ensure a thorough search. The Boolean search query used was (golimumab) AND (spondylitis OR spondyloarthritis) AND (paradoxical OR symptom flare-up OR induce OR induction) AND (inflammatory bowel disease OR colitis OR Crohn disease).

Two researchers (KAA and MA) independently evaluated all eligible articles and excluded duplicate and irrelevant ones. Additionally, we conducted a manual search and review of available references to ensure the inclusion of all possible cases.

Eligibility Criteria and Data Extraction

Paradoxical IBD in a patient with SpA was defined as the de novo development of any type of IBD without a history of the disease after receiving golimumab or the exacerbation of pre-existing IBD shortly after golimumab initiation.

The eligibility criteria included literature in the English language only, randomized controlled trials (RCTs), observational studies, meta-analyses, systematic reviews, case series/case reports, reviews, letters/correspondences, and original articles. The titles and abstracts were screened for relevance, focusing on identifying any cases linking IBD to golimumab as an adverse event. The exclusion criteria included cases without complete clinical descriptions, cases in which the diagnosis was ambiguous, unpublished abstracts and materials, book chapters, conference papers, and preprints.

We adhered to the guidelines outlined by the Preferred Reporting Items for Systematic Reviews and Meta-Analyses (PRISMA) [7]. Two independent researchers (KAA and MA) extracted detailed descriptions of the published cases, with any conflicts resolved by an arbitrator (AR).

Quality Assessment

We used an assessment tool to evaluate four quality domains, selection, ascertainment, causality, and reporting, of published case reports to reach an overall judgment for each case categorized as low, moderate, or high [8].

Results of Literature Search and Quality Assessment Results

**Figure 2:**
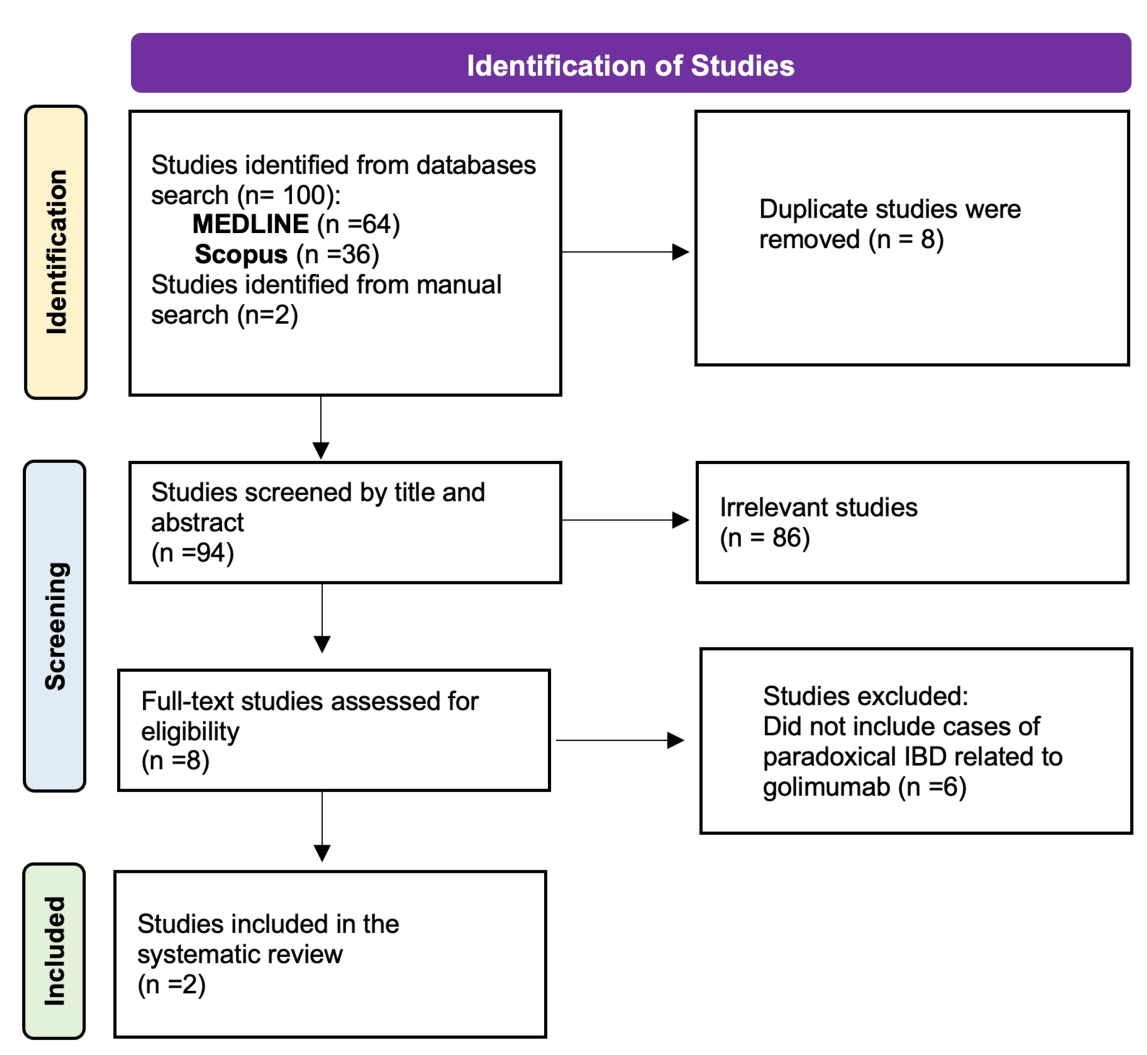
PRISMA search flow diagram (last search: October 9, 2024) PRISMA, Preferred Reporting Items for Systematic Reviews and Meta-Analyses; MEDLINE, Medical Literature Analysis and Retrieval System Online; IBD, inflammatory bowel disease

Figure 2 shows the flow diagram of the systematic literature review.

We identified four cases of paradoxical IBD following golimumab treatment for axSpA-related IBD, all in patients with a pre-existing diagnosis of IBD (CD, UC, and IBDU) (Table 2) [9,10].

**Table 2:** Clinical profiles of axial spondyloarthritis (SpA) patients with paradoxical IBD after golimumab (GOL) treatment F, female; M, male; CD, Crohn's disease; UC, ulcerative colitis; IBD, inflammatory bowel disease; AS, ankylosing spondylitis; GI, gastrointestinal; HLA-B27, human leukocyte antigen B27

Case number	Author(s) (year)	Age and sex	IBD history	Previous use of biologics	Approximate duration of GOL use before paradoxical IBD	Type of IBD and site	Treatment	Outcome	Quality evaluation
1	Fiehn and Vay (2011) [9]	47, F	CD before AS was diagnosed (positive HLA-B27)	Adalimumab	After two months	Flare (colonoscope- and biopsy-proven), terminal ileum	GOL discontinuation and local budesonide	Bowel symptoms resolved quickly	Selection, moderate; ascertainment, high; causality, moderate; reporting, moderate
2	Fiehn and Vay (2011) [9]	43, F	Axial SpA (positive HLA-B27) was diagnosed before CD	Infliximab, etanercept, and adalimumab	Approximately three months	Flare (colonoscope- and biopsy-proven), terminal ileum	GOL discontinuation and oral prednisolone. GOL was rechallenged, and azathioprine was started but resulting in the recurrence of CD requiring GOL discontinuation with oral prednisolone	GI symptoms slowly resolved	Selection, moderate; ascertainment, high; causality, high; reporting, moderate
3	Fiehn and Vay (2011) [9]	73, M	AS (positive HLA-B27) was diagnosed before nonspecific colitis	Adalimumab	After five months	De novo UC (biopsy-proven), pancolonic	GOL discontinuation and oral prednisolone	GI symptoms resolved	Selection, moderate; ascertainment, high; causality, moderate; reporting, moderate
4	Bawany et al. (2014) [10]	25, M	Ulcerative proctitis was diagnosed before AS (positive HLA-B27)	None	After three months	Flare (colonoscope- and biopsy-proven), rectum	Topical mesalazine dose was increased. GOL was discontinued. After one month, adalimumab was started due to partial response	Marked improvement in GI and back symptoms after a few weeks of starting adalimumab	Selection, moderate; ascertainment, high; causality, moderate; reporting, moderate
5	Our case	18, M	Juvenile AS (positive HLA-B27)	None	After two months	De novo IBD unclassified (colonoscope- and biopsy-proven) in all colon including the terminal ileum	GOL was discontinued after three doses. Celecoxib was restarted. Infliximab was commenced	Bowel symptoms improved significantly three weeks after stopping GOL	

Our case is the first to report paradoxical de novo IBD in a biologic-naïve AS patient who was HLA-B27-negative without a history of IBD symptoms. The clinical course of these reported cases was similar to that observed in our patient.

The clinical course of the four previously reported cases was similar to that observed in our patient. These cases involved patients with axSpA, all of whom were HLA-B27-positive with a prior diagnosis of IBD. In three cases, patients had been treated with TNF inhibitors (adalimumab, infliximab, or etanercept) before experiencing IBD worsening 2-5 months after initiating golimumab. One case involved a biologic-naïve patient with ulcerative colitis who developed IBD after starting golimumab.

Management strategies in these cases included discontinuing golimumab, rechallenging golimumab with the addition of azathioprine, increasing the dose of topical mesalazine, or switching to another TNF inhibitor. Gastrointestinal symptoms improved following these interventions.

Paradoxical reactions

Paradoxical reactions (PRs) were described in 1997 in chronic inflammatory systemic diseases and can occur in patients who are taking biologic or non-biologic drugs, regardless of the underlying disease activity [11].

IBD encompasses a group of idiopathic inflammatory diseases that primarily affect the GI system and predominantly comprise UC and CD and, to a lesser extent, IBDU or indeterminate colitis. IBDU has a prevalence of 10%-15% among all IBD, depending on the definition used. It may evolve into UC or CD or may remain unclassified [12,13]. Furthermore, subclinical gut inflammation as confirmed previously by ileocolonoscopy, demonstrating inflammation on biopsy, has been reported in 24%-49% of patients with AS. This inflammation, especially in chronic lesions, can progress to overt IBD in 6.5% of patients with SpA [14]. Golimumab treats axSpA and IBD specifically UC. Even if AS patients have coexisting IBD, it is expected to treat both diseases, which is not the case in our patient.

To explore the possibility of an association between golimumab and de novo IBD in our AS patient, we used the attribution analysis proposed by Miller et al., which includes four primary and three secondary elements [15]. The association between golimumab and IBDU in our patient appeared to fulfill four elements of attribution including three primary elements.

Firstly, there is a temporal relationship between the new onset of IBD and the administration of golimumab. Secondly, no alternative diagnoses were found. The main differential diagnoses in our patient were infectious colitis and celecoxib-induced colitis.

Patients who are on TNF-α inhibitors may be immunocompromised to a degree and are therefore at a higher risk of developing infections of the GI tract including parasites (e.g., *Cryptosporidium*) and enteric bacteria (e.g., *Salmonella*, *Shigella*, *Campylobacter*, and *Clostridium difficile*). The reactivation of latent tuberculosis (TB) can occur after initiating therapy with anti-TNF agents, often presenting with pulmonary manifestations, constitutional symptoms, and extra-pulmonary features. GI involvement constitutes 6% of the extra-pulmonary TB cases and typically affects the terminal ileum [16]. Despite the potential risk of opportunistic infections associated with TNF-α therapy, RCTs have not observed significant GI infections associated with golimumab therapy. In the Go-RAISE trial, no opportunistic infections were observed in patients with AS after treatment with golimumab for 104 weeks [17]. Additionally, there were no TB cases after five years of golimumab treatment [18]. Other studies, including extension studies of golimumab in RA and UC, have found only a few cases of TB reactivation [19-21]. In our patient, infectious colitis was ruled out based on history and negative tests for ova, parasites, and enteric bacteria, as well as the absence of respiratory symptoms, negative TB screening (via tuberculin skin test and biopsy), and normal chest X-rays. Although acute enterovirus infection was not ruled out by testing, it is unlikely given the chronic colitis observed on biopsy.

While the differential diagnosis in this patient included celecoxib-induced colitis, there is no evidence that NSAID use in patients with AS accelerates the progression of subclinical gut inflammation to overt IBD [14]. A study in patients with AS did not find a correlation between NSAID use and bowel symptoms or the elevation of fecal calprotectin (a marker of intestinal inflammation) [22]. In experimentally induced colitis, selective cyclooxygenase-2 (COX-2) inhibitors improve the severity of colitis [23]. A recent pooled meta-analysis found that selective COX-2 inhibitors are safe for the short-term treatment of musculoskeletal symptoms in most IBD patients compared to placebo. However, the careful monitoring of IBD flares is warranted during the first few days of treatment [24]. The GI symptoms in our patient were unlikely due to celecoxib because he had discontinued it two months prior to the onset of bowel symptoms, and he did not present with the typical features of NSAID-induced enteropathy, such as concentric diaphragmatic stricture, intestinal obstruction, anemia, or hypoalbuminemia [25].

Thirdly, our patient's IBD symptoms improved after the discontinuation of golimumab (dechallenge). Furthermore, he fulfills the fourth primary element (biologic plausibility). Alterations in the gut microbiome may induce local inflammation through an innate immune response. Although the exact mechanism of IBD development in patients with AS who are on TNF inhibitors is unknown, it may involve a cytokine imbalance caused by these drugs. In genetically predisposed patients, such as those with *NOD2*/*CARD15* gene mutations, TNF inhibition can activate autoreactive T cells, altering the inflammatory cytokine environment and leading to IBD. Additionally, since patients have a higher risk of IBD, paradoxical inflammatory conditions induced by TNF inhibition may unmask an underlying inflammatory disease [26-28].

Lastly, our patient's case is similar to the reported cases of other TNF inhibitors (analogy). Initially, numerous case reports and disease registries highlighted an association between new-onset IBD in patients with AS and JIA treated with TNF inhibitors, with etanercept being more frequently implicated than infliximab or adalimumab [27,29-40]. Subsequent RCTs and open-label trials in AS patients, however, found that the incidence of new-onset IBD was comparable to placebo in patients receiving TNF inhibitors, namely, infliximab, etanercept, and adalimumab [41].

A 2010 review of trials involving golimumab 50 mg monthly in RA, PsA, and AS showed that 3.3%-8.0% of patients developed non-bloody diarrhea, a rate similar to that of the placebo group. Importantly, no case of de novo IBD was diagnosed [42]. Furthermore, in a French nationwide series conducted between January 2009 and December 2010, 16 patients were diagnosed with new-onset IBD following treatment with TNF inhibitors, including etanercept, adalimumab, and infliximab. Of these, 12 patients had AS, while the remaining patients had PsA, RA, or JIA with enthesitis-related arthritis. Etanercept was the primary agent responsible for over 80% of these cases. The types of IBD observed included CD (eight patients), Crohn's-like disease (six patients), and IBDU and UC (one patient each) [28]. In the Go-RAISE study, which spanned a five-year period of golimumab use in patients with AS, there were no documented cases of new-onset IBD [18].

In other inflammatory conditions, including psoriasis and PsA, both the exacerbation of existing IBD and the emergence of new cases induced by TNF inhibitors have been documented. Notably, CD occurs in 50% of cases and Crohn's-like disease in 37.5%, and UC and IBDU each occur in 6.3% of cases [43].

A recent retrospective study found that 10 out of 154 patients with AS who received TNF inhibitors (infliximab, etanercept, adalimumab, and golimumab) developed new-onset IBD. None of these cases occurred with golimumab, and no significant differences were detected among the three other TNF inhibitors. This study also found a higher incidence of new-onset IBD in patients receiving TNF inhibitors than in those who did not receive this treatment. Therefore, it was concluded that paradoxical IBD induced by TNF inhibitors should be considered [44].

Recently, the British Society for Rheumatology Biologics Register in Ankylosing Spondylitis (BSRBR-AS) did not find any new IBD cases associated with golimumab. Additionally, a meta-analysis of RCTs and observational studies confirmed this finding up to one year after initiating golimumab treatment [45].

Based on published case reports and disease registries, several management options are available for addressing IBD as a paradoxical reaction to TNF inhibitors. These include discontinuing the offending TNF inhibitor, which is often the first step in management. Alternatively, switching to a different TNF inhibitor, excluding etanercept, is a widely used approach. In less common scenarios, continuing the offending drug while using other immunosuppressive therapies, such as local corticosteroids, azathioprine, mesalazine, sulfasalazine, or methotrexate, has been shown to yield favorable outcomes [28,43]. Additionally, oral JAK inhibitors, such as tofacitinib and upadacitinib, are also treatment options. Both agents are approved for managing AS and UC, with upadacitinib also indicated for non-radiographic axSpA and CD [1,46].

Our case report has some limitations. As a single retrospective case, it inherently limits generalizability. The absence of a baseline colonoscopy prior to golimumab initiation prevents the definitive establishment of causality between golimumab and IBD onset. Furthermore, although our literature review was comprehensive within major medical databases, we acknowledge that additional relevant cases might exist in other sources that were not searched.

## Conclusions

This case report is the first in the literature to describe paradoxical de novo IBD induced by golimumab in a biologic-naïve, HLA-B27-negative AS patient without prior IBD symptoms. This highlights the need for clinicians to remain vigilant for GI symptoms in AS patients initiating golimumab, even in those without known IBD. Early recognition and prompt referral to a gastroenterologist are essential for timely diagnosis and co-management.

While this case suggests that golimumab may rarely contribute to paradoxical IBD, it underscores the importance of evaluating anti-TNF agents individually, as previous reports have implicated etanercept more commonly than infliximab or adalimumab. Our findings expand the current understanding of golimumab's safety profile in AS treatment. Although attribution analysis was used to assess the association between golimumab and IBD onset, causality cannot be definitively established. Larger studies are needed to clarify the risk of paradoxical IBD with golimumab compared to other anti-TNF agents and to identify potential patient-specific risk factors.
